# Role of Cellular Metabolism during *Candida*-Host Interactions

**DOI:** 10.3390/pathogens11020184

**Published:** 2022-01-28

**Authors:** Aize Pellon, Neelu Begum, Shervin Dokht Sadeghi Nasab, Azadeh Harzandi, Saeed Shoaie, David L. Moyes

**Affiliations:** 1 Centre for Host-Microbiome Interactions, Faculty of Dentistry, Oral & Craniofacial Sciences, King’s College London, London SE1 9RT, UK; neelu.begum@kcl.ac.uk (N.B.); shervin_dokht.sadeghi_nasab@kcl.ac.uk (S.D.S.N.); azadeh.1.harzandi@kcl.ac.uk (A.H.); saeed.shoaie@kcl.ac.uk (S.S.); 2 Science for Life Laboratory, KTH—Royal Institute of Technology, SE-171 21 Stockholm, Sweden

**Keywords:** immunometabolism, metabolism, macrophages, epithelial cells, glycolysis, glucose, moonlighting proteins, *Candida albicans*

## Abstract

Microscopic fungi are widely present in the environment and, more importantly, are also an essential part of the human healthy mycobiota. However, many species can become pathogenic under certain circumstances, with *Candida* spp. being the most clinically relevant fungi. In recent years, the importance of metabolism and nutrient availability for fungi-host interactions have been highlighted. Upon activation, immune and other host cells reshape their metabolism to fulfil the energy-demanding process of generating an immune response. This includes macrophage upregulation of glucose uptake and processing via aerobic glycolysis. On the other side, *Candida* modulates its metabolic pathways to adapt to the usually hostile environment in the host, such as the lumen of phagolysosomes. Further understanding on metabolic interactions between host and fungal cells would potentially lead to novel/enhanced antifungal therapies to fight these infections. Therefore, this review paper focuses on how cellular metabolism, of both host cells and *Candida*, and the nutritional environment impact on the interplay between host and fungal cells.

## 1. Introduction

Fungal microorganisms inhabiting the human body, namely the mycobiota, constitute an essential part of the microbiota, despite their relatively low number compared to their bacterial counterparts [1,2]. Commensal fungi, either being permanent or transient colonisers, populate the skin and mucosae covering the oral cavity and the respiratory, gastrointestinal, and genitourinary tracts. Unsurprisingly, different genera governing each body site, including *Candida* (oral cavity and gut), *Malassezia* (skin), *Saccharomyces* (gut) or *Eremothecium* (lung) [3,4,5]. Remarkably, many of these genera, as well as other species present in our environment, are pathobionts, capable of becoming pathogenic when host immunity or tissue microenvironment changes.

Among fungal pathogens, *Candida* spp., and specifically *C. albicans*, remain the most clinically relevant fungi, causing a wide range of infections in humans from superficial to systemic candidiasis [6]. The emergence of antifungal drug resistance in *C. albicans*, as well as the increasing prevalence of infections by other *Candida* species that are intrinsically resistant to available drugs (e.g., *Candida auris*) [7], highlights the importance of finding novel therapeutic strategies to deal with these infections.

In the last couple of decades, the importance of the nutritional environment and metabolism of both host and pathogens during infectious processes has been highlighted [8]. The presence or abundance of certain metabolites, including simple carbohydrates such as glucose or galactose, modulates cellular responses of both pathogen and host, therefore being essential factors during their interactions. The stress derived from the interaction with the other organisms often leads to metabolic reprogramming that supports immune responses on one side and pathogenic/commensal growth on the other.

This review paper aims to explore the current knowledge regarding the role of host metabolism in the control of innate immune responses to fungal microbes on one side, and the importance of *C. albicans* metabolism for commensalism and virulence on the other. We will also highlight how metabolism modulates the biology of both host and fungal cells during their interactions, and the emerging strategies to develop novel therapeutic tools.

## 2. Immunometabolism: Feeding Immune Responses in the Host

During the last two decades, an increasing body of evidence has identified the key role of cellular metabolism in developing immune responses, either enhancing (contributing to pathogen clearance) or diminishing (contributing to tolerogenic states) them. Thus, a new research field termed immunometabolism developed to delve into the control of immunity driven by metabolic processes [9]. Metabolic regulation of immunity has been described in both adaptive (e.g., T cells) [10] and innate (e.g., macrophages) [11] cells. Both types of cells show a wide spectrum of metabolic profiles upon activation with different stimuli. Since host immunometabolism has been extensively reviewed in recent years, we will focus on innate immunity, giving a general overview of how metabolic reprograming occurs and the modulation of immune responses by metabolites and metabolic enzymes.

### 2.1. Metabolic Reprogramming in Immune and Non-Immune Cells

Interaction of innate immune cells, such as macrophages and monocytes, with different microorganisms leads to metabolic shifts on which their responses rely. These responses are either boosted or decreased to promote infection clearance or microbial tolerance, respectively. Since there is a great diversity of microbial structures (e.g., pathogen-associated molecular patterns (PAMPs)) and of host receptors (pattern recognition receptors (PRRs)) involved in their detection, the metabolic profiles of these differently stimulated cells, along with their derived immune responses, are also very diverse [12]. 

**Figure 1:**
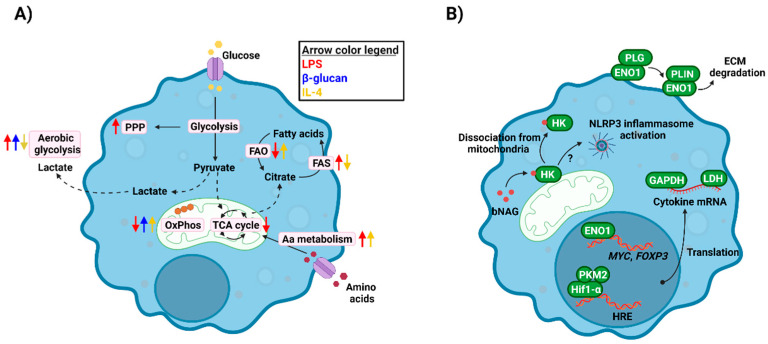
Immunometabolism in innate immune cells. (**A**) Challenging innate immune cells with either exogenous (LPS, β-glucan) or endogenous (IL-4) stimuli leads to metabolic reprogramming, which involves changes in pathways as glycolysis, FAO/FAS, OxPhos, etc. These shifts in metabolism provide cells with energy and building blocks to develop their functions. (**B**) Metabolic enzymes regulate immune responses at many levels, including their moonlighting functions. They can act as transcription/translation facilitators (ENO1, PKM2, GAPDH, LDH), immune receptors or activators (HK) or facilitators of immune cell migration (ENO1). bNAG, bacterial N-acetylglucosamine; ECM, extracellular matrix; ENO1, enolase 1; FAO, fatty acid oxidation; FAS, fatty acid synthesis; GAPDH, glyceraldehyde dehydrogenase; HK, hexokinase; LDH, lactate dehydrogenase; OxPhos, oxidative phosphorylation; PKM2, pyruvate kinase M2; PLG, plasminogen; PLIN, plasmin; PPP, pentose phosphate pathway; TCA, tricarboxylic acid. Created with BioRender.com (last accessed 12 January 2022).

Alterations in glucose uptake and metabolism are the main hallmark of metabolic shifts in innate immune cells (Figure 1A). For instance, when macrophages are challenged with bacterial lipopolysaccharide (LPS), glucose uptake and processing via aerobic glycolysis increases, that is glycolysis coupled with lactate dehydrogenase activity leading to lactic acid production in normoxic conditions. Conversely, there is decreased activity in the tricarboxylic acid (TCA) cycle and oxidative phosphorylation (OxPhos) [13]. In contrast, cells stimulated with fungal β-glucan show an increase in both aerobic glycolysis and OxPhos [14]. Shifts in cellular metabolism towards aerobic glycolysis provide cells with more rapid energy and building blocks generation, and leading to increased cytokine release, etc. [15]. In contrast, anti-inflammatory macrophages rely on aerobic respiration, completely oxidising glucose through glycolysis, the TCA cycle and OxPhos [9].

Besides glucose metabolism, other pathways are involved during metabolic reprogramming of innate immune cells [9,16]. These pathways provide energy or redox molecules, or intermediate metabolites serving as building blocks or having regulatory functions, as explained below. The pentose phosphate pathway (PPP) provides proliferative cells with metabolites needed for nucleotide synthesis, but also contributes to NADPH production. Notably, this pathway is upregulated after LPS activation of macrophages [17], which has been related to the increased reactive oxygen species (ROS) generation in these cells via NADPH oxidase [18,19]. Fatty acid synthesis (FAS) or oxidation (FAO), alongside other lipid metabolism pathways, are also differentially regulated in activated macrophages [20]. Pro-inflammatory cells use FAS and citrate accumulated due to the TCA cycle shut down to synthesise fatty acids, prostaglandins, and leukotrienes, essential molecules for signalling events, inflammation, etc. [20,21]. In contrast, since anti-inflammatory macrophages keep their TCA and OxPhos intact but lower glycolytic levels, they rely on FAO and fatty acid uptake to feed those pathways. Finally, amino acid metabolism, such as glutamine or arginine, is key for innate immune responses, including nitric oxide production or cytokine production [9].

A number of signalling pathways govern metabolic shifts in innate immune cells [22]. Among them, the activation of the transcription factor hypoxia-inducible 1α (HIF-1α) is involved in the increase in glycolytic activity observed in LPS-activated macrophages and is responsible for the expression of several immunity-related genes [23,24]. Similarly, signalling via mechanistic target of rapamycin (mTOR) is involved in promoting cholesterol and FAS, as well as sensing amino acid and glucose availability [25].

In recent years, the ability of innate immune cells to develop long-term responses has been described, adding more complexity to the biology of these types of cells [26,27]. Essentially, the term “innate immune memory” involves a wide range of phenotypes mainly observed in monocytes/macrophages that renders them more tolerogenic or reactive against a second encounter [28]. Notably, these events are intimately linked to epigenetic and metabolic reprogramming of cells [29,30], both of which are the consequence of signalling promoted by the first encounter with the microbial challenge. 

Since the discovery by Otto Warburg of the metabolic shift towards aerobic glycolysis undergone by some cancer cells [31], metabolic reprogramming has been observed in many cell types, especially in a pathologic context such as cancer. Although much of the work on these shifts driven by microbes has been carried out on immune cells, non-immune cells playing paramount roles during the infectious processes undergo similar shifts. Viral infections have been shown to modulate metabolic profiles of airway epithelial cells [32] and endothelial cells [33]. Similarly, the murine bacterial pathogen *Citrobacter rodentium* promotes a decrease in carbohydrate metabolism in intestinal epithelial cells [34], whilst skin keratinocytes increase their aerobic glycolytic metabolism in response to *Staphylococcus aureus* [35]. Despite this, the consequences of these metabolic shifts on immune responses developed by these cell types are yet to be further explored.

### 2.2. Beyond Metabolic Reprogramming: Immune Regulatory Roles of Metabolic Enzymes and Metabolites

Besides the direct impact of metabolic reprogramming on immune cell activity (e.g., energy and redox balance, metabolite catabolism/anabolism, etc.), there are other levels of regulation of immune responses in which metabolic enzymes or metabolites play a role (Figure 1B). 

Some metabolic enzymes have been observed to display regulatory functions distinct from their metabolic activities. Therefore, these proteins have been termed as “moonlighting proteins”. These alternative functions of metabolic enzymes can be found among diverse biological organisms and were firstly observed in microorganisms, including bacteria and fungi, such as *Candida* spp., in which they have roles in microbial cell adhesion, pathogenicity, etc. [36,37]. Notably, the capacity of these proteins to develop moonlighting functions has been conserved in mammalian cells [38]. 

Glycolytic enzymes, such as hexokinase, glyceraldehyde-3-phosphate dehydrogenase (GAPDH), or enolase, display these moonlighting functions in very different ways. Hexokinase, the enzyme catalysing the first step in glycolysis, is one of the main proteins upregulated upon cell activation, and its inhibition by 2-deoxyglucose (2-DG) leads to a significant reduction in pro-inflammatory marker release [17]. However, hexokinase was recently described as a new intracellular pattern recognition receptor able to bind to the bacterial peptidoglycan component N-acetylglucosamine. This binding leads to hexokinase separation from mitochondria and drives NLRP3 inflammasome activation [39]. GAPDH regulates cytokine release in both T cells [40] and monocytes [41] by directly binding to cytokine mRNA. Enolase, involved in one of the final steps of glycolysis, has been associated with monocyte binding to plasminogen, facilitating their migration [42]. Moreover, this enzyme can modulate gene expression, including *MYC* [43] and *FOXP3* [44], by directly binding to gene regulatory elements. Similarly, PKM2 (pyruvate kinase isoform M2) can act as a co-activator of Hif-1α and regulates IL-1β expression through the activation of NLRP3 and AIM2 inflammasome [45,46]. Finally, lactate dehydrogenase (LDH), the last enzyme in the aerobic glycolytic pathway converting pyruvate in lactate, is able to bind to cytokine transcripts to modulate their translation [47,48].

It is not just proteins/enzymes involved in metabolic processes that can have these alternative immune functions. Metabolites derived from central metabolic pathways, both intermediates and final products, have been shown to modify protein function/structure and in that way modulate immune cell biology. The best-described process by which metabolites regulate immune responses is via protein post-translational modifications (PTMs). These modifications are of special relevance in the case of histones as they lead to changes in the expression of a wide range of genes—the field of epigenetics. These histones PTMs are manifold, including acetylation, phosphorylation, deamination, and methylation among others. Of these, lysine acetylation is one the clearest examples of the link between metabolism and cell functions. Acetyl-CoA is a key metabolite used by lysine acetyltransferases as a donor to acetylate proteins, although this process can occur non-enzymatically [49]. Moreover, acetyl-CoA intracellular levels correlate with protein acetylation rates and thus, changes in the nutritional environment of cells or tissues are associated with changes in acetylation levels [50].

Besides acetylation, a great variety of histone PTMs associated with metabolism has been described to date, most of them involving short-chain fatty acids (SCFAs) such as propionate, butyrate, crotonate or succinate [51]. This process is thus tightly regulated by cellular metabolism and the nutritional environment since the level of each histone acylation depends on the concentration of their respective acyl-CoA [52]. Notably, many of these PTMs have been discovered very recently and novel forms are predicted to be found in the near future. In fact, histone lysine lactylation was recently described in both human and mouse cells [53]. The event was regulated by exogenous glucose, hypoxia, and glycolytic activity levels, all three being positively correlated with intracellular lactate levels. Specifically looking at macrophages, the authors showed that stimulation of M1 polarisation using an acute LPS and interferon-γ challenge led to higher lactate production because of the expected shift towards aerobic glycolysis. Coupled RNA-seq and lactylation-specific ChIP-seq analyses of activated macrophages showed the modulation of gene expression by these PTMs, with pro-inflammatory genes being regulated at early timepoints whilst M2 profile-related gene expression was modulated during a later phase. This suggests that histone lactylation sets a gene expression “timer” that leads to homeostasis after the inflammatory burst [53]. 

Metabolic intermediates can also act as intra- or extracellular signals to modulate immune responses via mechanisms beyond epigenetic modifications [54,55]. The proven existence of a wide range of metabolite transporters [56] and receptors [57] has shown the potential impact of their availability on immune cell biology. Metabolite transporters facilitate metabolite uptake and secretion, highlighting the paramount relevance of the nutritional microenvironments created during, for example, inflammatory processes. The second, metabolite receptors, are usually G-protein-coupled receptors sensing metabolites and triggering intracellular signalling events, which has led to the hypothesis of some metabolites having cytokine/chemokine-like functions [54]. Moreover, some of these metabolites, such as lactate [58] or succinate [17], have been associated with functional stabilisation of such relevant proteins as HIF-1α, the master regulator linking metabolism to immunity.

## 3. Candida Metabolism: The Significance of Being Adaptable

As commensals and opportunistic pathogens, *Candida* spp. have developed high degree of phenotypic plasticity to adapt to diverse and changing environments. Therefore, metabolism is an essential part of *Candida* survival for nutrient assimilation and pathogenicity. The virulence of *C. albicans* is related to gene expression and host immune status [59]. *Candida* genes encoding metabolic enzymes directly interact with the host mediating fungal virulence. These virulence mechanisms include yeast-hyphal morphogenesis, phenotypic switching in the opacity of cells, adhesion, secreted hydrolases, and moonlighting proteins. *Candida* metabolic flexibility and evolution emphasises the challenges in investigating metabolic divergency with particular attention to clinical and therapeutic intervention [60,61].

Carbon assimilation and its accompanying metabolic pathway plasticity has been widely explored in *C. albicans* [62]. The carbon metabolic framework, including glycolysis, the TCA cycle and gluconeogenesis, is controlled by regulatory networks based on local nutrient availability. Metabolic plasticity allows *C. albicans* to assimilate glucose and other carbon sources simultaneously, unlike *S. cerevisiae* that switches to fermentative pathway in the presence of glucose [63]. This confers fitness in survival and adaptation to *Candida* in different host niches. General control of amino acid metabolism (GCN response) has also been linked to pathogenicity and virulence attributes of *Candida* species [61,64].

### 3.1. Impact of Metabolism on Fungal Biology: From Morphogenesis to Cell Wall Synthesis

**Figure 2:**
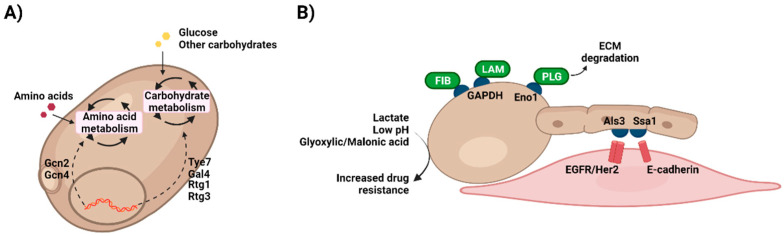
Metabolic plasticity in *Candida*. (**A**) *Candida* species are able to grow on a wide range of compounds, giving them the chance to thrive in very different environments. This metabolic plasticity is tightly regulated by a network of transcription factors that are activated depending on the nutritional requirements of the fungus. (**B**) Growing on different compounds leads to changes in *Candida*, for example cell wall structure or composition. This is of special importance when physiologically relevant nutrients, such as lactate, are present. Utilization of these metabolites by *C. albicans* remodel its cells wall increasing antifungal drug resistance. Moreover, like host cells metabolic enzymes in *Candida* display moonlighting functions associated with, for instance, cell adhesion to the ECM (GAPDH, Eno1) or host cells (Ssa1). ECM, extracellular matrix; Eno1, enolase 1; GAPDH, glyceraldehyde dehydrogenase; FIB, fibronectin; LAM, laminin; PLG, plasminogen. Created with BioRender.com (last accessed 12 January 2022).

*C. albicans* displays a remarkable metabolic plasticity, being able to grow in the presence of different carbon sources, such as glucose, fructose, or galactose (Figure 2A). However, it shows preference towards the first one and in fact, growing on glucose as the only carbon source allows the fungus to thrive in the presence of a wide range of nutritional and stress conditions [65]. The transcriptional regulators Tye7 and Gal4 are key for the catabolism of glucose and other hexoses by *C. albicans*, controlling the expression of genes involved in glycolysis, fermentation, pyruvate dehydrogenase complex (Gal4 only), or trehalose metabolism (Tye7 only) [66]. Furthermore, Tye7 assists in cohesiveness and hyphal formation in biofilms although its absence does not impact on hyphal growth in planktonic conditions [67]. Defects in Tye7 function do not have a great impact on systemic candidiasis but have a significant effect on *C. albicans* ability to colonise the gut [68]. Gal4 regulates a unique set of carbohydrate genes initiated in hypoxic conditions that are essential for pathogenicity. Fermentable carbon sources such as galactose enhance the glycolytic pathway and minimise dependency on fermentation [66]. Interestingly, two Gal4 analogues, Rtg1 and Rtg3, have a great impact during both systemic infections and gut colonisation, although they are involved in the regulation of a broader range of cellular processes [68]. 

Moreover, carbohydrate metabolism is intimately linked to *C. albicans* morphogenesis, with nutrient starvation or serum presence being among the factors inducing the yeast-to-hypha transition [69,70]. Metabolic genes are regulated during hyphal growth, including Adh1, Pgk1, and Gpm1 [71]. Similarly, white and opaque *Candida* cells display different metabolic profiles, with the white phenotype being more fermentative and the opaque being more oxidative and using FAO [65]. In fact, metabolic genes including Eno1, Fba1, Pyk1, Tpi1 and Pgi1 [72], are regulated by the central morphogenetic regulator Efg1, a transcription factor related to the white-opaque transition. Efg1 expression appears to be mechanistically connected to carbon metabolism in *Candida*. In general, Efg1 is downregulated in fermentative metabolism and upregulated in oxidative metabolism involved in morphogenesis [59,73,74]. Moreover, Efg1 stimulates fermentation and suppression of respiratory metabolism, demonstrating the importance of glycolytic metabolism in controlling virulence attributes [73]. This ability allows *Candida* species to switch between opaque and white cells (fermentative metabolism) depending on the nutritional environment [74]. 

As well as glycolysis, other metabolic pathways have an impact on *Candida* virulence. Knockout of FAO, for example, does not prevent candidiasis but assists in systemic virulence [75,76]. Conserved GCN networks, including *GCN4* and *GCN2* genes, are vital regulators activated during amino acid starvation. They act to reduce protein translation rates and induce cellular morphogenesis in *C. albicans* [77]. *GCN4* is a master regulator that activates morphogenesis via the Ras-cAMP signalling pathway to form pseudo-hyphae and activating amino acid biosynthetic genes [77]. In addition, GCN, particularly upregulation of *GCN4* gene, is further required for efficient biofilm formation in *C. albicans* [78]. On the other hand, the arginine pathway, meanwhile, appears to be essential in *C. albicans* as mutations in this pathway caused a defect in germ tube and hyphal formation [79]. Finally, the amino sugar N-acetylglucosamine (GlcNAc), which is the main component in chitin within the fungal cell wall, stimulates cellular responses mediating virulence, comprising of yeast-hyphae transition and stress responses [80]. 

The cell wall protects fungal cells from the environmental stress, controls cell morphogenesis, allows for immune recognition and is essential for cell growth of *Candida* species [81]. *C. albicans* uses sugars such as glucose, mannose, and galactose to provide energy to synthesise the cell wall. Thus, metabolic regulation is important in cell wall remodelling with the main constituents being β-glucan, chitin, and an outer layer consisting of mannoproteins (mannosylated proteins) [82]. The generation of these cell wall components requires glucose via both glycolysis catabolic and gluconeogenesis biosynthetic pathways [83]. The relative proportions of these components in the cell wall changes depending on the cells’ environment. For example, β-glucan in cells within biofilms is elevated compared to non-biofilms [84]. The use of carbon sources alternative to glucose have been attributed to differences in cell wall architecture, adherence, biofilm formation, resistance to antifungal drugs and responses to stress [59,85,86]. For instance, the growth of *C. albicans* on lactate led to cell wall restructuring leading to increased resistance to azoles and oxidative stress. However, fungal cells grown on lactate media showed increased pores, higher hydrophobicity, and less elastic cell walls with reduced thickness of β-glucan and chitin [85].

### 3.2. Impact of Metabolism on Candida Pathogenic Potential

As discussed above, *Candida* species have a robust metabolism that contributes to virulence factors (Figure 2B). Like host cells, *Candida* invasion strategies include moonlighting proteins with distinct functions. These multifunctional proteins perform additional actions to their canonical biochemical function [87,88]. Owing to evolution, some moonlighting proteins can display their different functions simultaneously, whilst others alter their activity or cellular location in response to environmental changes and cell survival needs [88]. To survive within different environments in the host organism during disease progression, microbes need to use adaptable mechanisms other than common virulence features, such as adhesion molecules and hydrolytic enzymes. In *Candida* species, different moonlighting proteins can be found attached to the cell wall, and they enable microbial cells to be more flexible and adaptable in a dynamic host environment during colonisation and invasion [89]. GAPDH, usually present in the cytoplasm, may be localised in the cell surface of *C. albicans* where it facilitates cell adhesion to fibronectin and laminin, hence helping the fungal attachment to the host and initiation of candidiasis [90]. Similarly, enolase has been identified in the surface of several clinically relevant fungi, with this enzyme being involved in fungal cell adhesion via plasminogen binding (as with macrophages) and in the degradation of the extracellular matrix (ECM) [91,92]. Moreover, the intracellular chaperone Ssa1, a member of the heat shock protein 70 family, has been shown as another atypical protein with localisation in *C. albicans* cell wall. This moonlighting protein also plays a key role during colonisation of host cells as an adhesin, acting jointly with Als3 to bind to EGFR/Her2 and E-cadherin [93,94,95].

While for decades there have been well-known classes of anti-fungal drugs, some of them do not specifically target fungi, hence showing toxicity for mammalian cells. Therefore, there is an urgent need to develop novel drug strategies [96]. As mentioned previously, *Candida* cell functions, such as cell wall construction and adaptation to environmental stress, significantly rely on nutrient availability and the type of carbon source. Equally, antifungal drug resistance can be also modulated by the nutritional environment. Deficit of glucose as the main carbon source force *C. albicans* cells to find an alternative source and therefore changes in the downstream machinery pathways, which could result in adaptation of the cell against different stress. Previously, it has been observed the *C. albicans* growth in presence of fermentable substrate, glucose, and non-fermentable, lactate, can change cell secretome, as well as alter the cell wall structure and proteome [97,98]. These modifications affect resistance to antifungal drugs and susceptibility to stress. In fact, *C. albicans* grown in the presence of lactate was more resistant to amphotericin B, caspofungin, and tunicamycin, whilst it showed increased susceptibility to miconazole [85]. In addition to alternative carbohydrate sources, the acidity of the environment can also make *Candida* susceptible to antifungal drugs. Growing *C. albicans* under vaginal simulated media and in the presence of acetic acid rendered it more susceptible to fluconazole. However, *C. albicans* susceptibility to fluconazole remained unchanged when some other organic acids, such as glyoxylic acid and malonic acid, were present [99]. Similarly, *C. glabrata* shows higher susceptibility in the presence of acetic acid compared to when it is just grown on glucose [100]. These examples show the importance of carbon source availability and elucidation of the role of different nutrient in the *Candida* pathogenicity and antifungal resistance, and the need for more in depth and targeted metabolic analysis on the drug efficacy to tackle the resistome problem in fungal infections.

## 4. The Role of Metabolism during Host-*Candida* Interactions

Interactions of *C. albicans* with innate immune and epithelial cells have been extensively studied in the past [101,102]. Great strides have been made in our understanding of how host cells recognize this fungus via PRRs, although the relevance of each receptor varies depending on the infection context, either being systemic [103] or at the mucosal barriers [104]. Phagocytosis by immune cells [105], or attachment to and invasion of epithelia [29], are the next steps in the infectious process and are essential to promote immune responses in these cell types. Secretion of the peptide toxin candidalysin contributes to cell damage and activation, especially in the case of epithelial cells [106,107].

**Figure 3:**
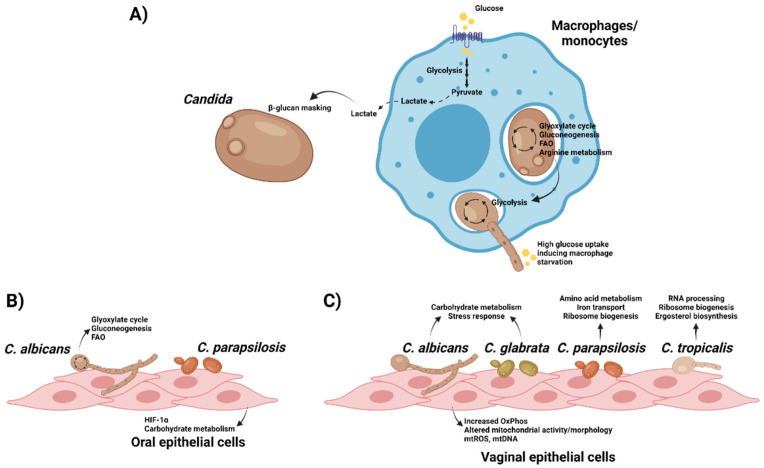
Interplay between *Candida* and host cells lead to metabolic reprogramming in both organisms. (**A**) Recognition of *C. albicans* by macrophages/monocytes drive changes in metabolism towards aerobic glycolysis, leading to the production of lactate that can be used by the fungus to enhance its β-glucan masking to evade immune responses. In turn, phagocytosed fungal cells use their metabolic plasticity to adapt to the nutrient-poor environment inside phagolysosomes. After piercing cell membranes using hyphae, *C. albicans* switches to glycolysis and depletes glucose from the medium, leading to macrophage cell death. (**B**) Oral epithelial cells activate HIF-1α when challenged with *C. parapsilosis*, whilst *C. albicans* adapts its metabolism upon interaction with these cells. (**C**) Variable responses are observed in different *Candida* species after interacting with vaginal epithelial cells. However, host cells exert a common early response to all of them mediated by changes in mitochondrial activity and morphology, with higher release of mitochondrial reactive oxygen species (mtROS) and DNA (mtDNA). Created with BioRender.com (last accessed 12 January 2022).

However, there are still a lot of gaps in our knowledge of how all these responses are regulated. As explained above, host cells undergo metabolic reprogramming upon interacting with microbes or microbial components, modulating how they respond to infections and competing over nutrients. In this section, we will discuss the current knowledge regarding the role of (immuno)metabolism during fungal interactions with epithelial and innate immune cells (Figure 3).

### 4.1. Impact of Metabolism during C. albicans Interactions with Immune Cells

Following their first contact with *C. albicans* (i.e., recognition, phagocytosis, etc.), activated immune cells reprogram their metabolism to mount an effective response. Transcriptomics-based analysis of peripheral blood mononuclear cells (PBMCs) stimulated with *C. albicans* shows a consistent upregulation of glycolysis, whilst no change (TCA cycle) or even downregulation (PPP) was observed for other pathways [108]. Specific stimulation of monocytes by heat-killed yeast or hyphae drives upregulation of several glycolytic enzymes, along with increased lactate production and glucose consumption, suggesting a shift towards aerobic glycolysis. Like β-glucan-stimulated cells [14], heat-killed cells promoted both higher ECAR (extracellular acidification rate) and OCR (oxygen consumption rate) levels, showing that OxPhos is also upregulated. This increased glycolysis plays a key role in immune responses as inhibiting glycolysis (2-DG and dichloroacetate, DCA) and mTOR pathway signalling (Torin1) significantly downregulates cytokine production post-fungal challenge [108].

The induced shift in metabolic pathways of infected monocytes with *C. albicans* differs between yeast and hyphal stimulation and is mediated by C-type lectins (CLR) but not by Toll-like receptors (TLR), showing the heterogenicity of host receptors in fungal recognition and responses. The responses generated to different *Candida* morphotypes is also varied. Monocytes infected with yeast cells activate glycolysis, oxidative phosphorylation, and glutaminolysis, whilst those infected with hyphae activate only glycolysis. Thus, we can see that the mechanisms of glucose metabolism are central players in regulating anti-*C. albicans* immunity and cytokine production [108]. Similarly, *A. fumigatus* induces an increase in aerobic glycolysis that is involved in macrophage responses to this filamentous fungus [109]. Of note, induction of metabolic reprogramming is mediated by the phagosomal removal of *A. fumigatus* melanin and its detection by the recently discovered melanin receptor MelLec [110]. This recognition is involved in HIF-1α mobilisation and subsequent cytokine release [109].

The main interface of host-fungal interaction is the fungal cell wall, a highly flexible structure with ability to remodel itself in the presence of a variety of environmental pressures, such as antifungal drugs [111]. Immune cells activated by *C. albicans* infection generate metabolites that can be sensed by fungi, which then remodel their cell wall in response to improve their immune evasion/protection. The major component of fungal cell walls, β-glucan, is a major fungal PAMP involved in the activation of many of the host antifungal responses [112]. The increased lactate levels associated with the shift to aerobic glycolysis may lead to β-glucan masking, preventing recognition of this key PAMP [113,114]. This phenotype (observed in multiple pathogenic *Candida* species) is only activated by appropriate lactate concentrations, and not by other metabolites such as proline, acetate, and methionine. This phenomenon is facilitated by the activation of the Crz1 transcription factor by the G protein-coupled receptor, Gpr1. Crz1 modulates the expression of genes involved in lactate-induced β-glucan masking. The outcome of this masking is significantly reduced visibility of *Candida* cells in terms of immune responses and thus diminished levels of tumour necrosis factor-alpha (TNFα) release and neutrophils [113]. Thus, *Candida* can successfully escape from macrophage uptake by taking advantage of the carbon sources released during metabolic rewiring of host cells in response to infection.

Upon *C. albicans* infection, macrophages are recruited to the site of infection and engulf fungal cells to try to destroy them or inhibit their growth in the phagolysosome through oxidative and nitrosative mechanisms [115]. *C. albicans*, however, has developed mechanisms to survive inside macrophages through metabolism manipulation. Two successive reprograming events of macrophages in response to *Candida* have been identified as follows, using whole-genome arrays: the early and late responses [116]. Their transcriptional profiles show the enhancement of gluconeogenesis and the glyoxylate cycle by upregulation of all genes involved in the conversion of fatty acid to glucose and a massive down-regulation of translation-related genes during the early response, suggesting a switch from glycolysis to gluconeogenesis, the glyoxylate cycle, and FAO during this early response. In addition, *Candida* cells phagocytosed by either macrophages [116] or neutrophils [117] upregulate arginine biosynthetic genes in response to ROS, rather than nutrient starvation, with these genes being important for germ tube and hyphal formation [118]. In contrast, the late response includes the reactivation of protein translation machinery and glycolysis. The metabolic shift in *C. albicans* cells following interaction with macrophages is assumed to be driven by the poor nutrient availability inside macrophage phagolysosomes, in which glucose concentration for instance is extremely low. This metabolic remodelling is dependent on the pathogenicity of *C. albicans* and *C. glabrata* since the non-pathogenic fungus *Saccharomyces cerevisiae* fails to demonstrate this response [76,116,119,120,121]. Moreover, similar events occur in *C. albicans* when it is exposed to neutrophils or whole human blood [117,120,122].

As stated earlier, during infection, activated macrophages shift their metabolism to aerobic glycolysis to activate antimicrobial inflammation and host defences. This means that for their survival *Candida*-activated macrophages rely specifically on glucose as their carbon source and additionally cannot reactivate mitochondrial oxidative phosphorylation. At the same time, ingested *C. albicans* cells similarly switch to aerobic glycolysis in the later phase of infection. As a result, macrophages and their ingested *C. albicans* compete for the available glucose [123]. During this nutrient war, the combatants rapidly consume the local glucose, leading to glucose depletion and triggering the “starvation” death of macrophages. Unlike macrophages, *C. albicans* cells have enough metabolic plasticity to switch their carbon source to alternatives such as the glyoxylate pathway, and in doing so survive the loss of glucose. As described earlier, these events are regulated by Tye7 and Gal4 *C. albicans* transcription factors. In *tye7Δ/Δgal4Δ/Δ* mutant strains, glycolysis and glucose consumption occurs at a far lower rate and, therefore, induction of macrophage starvation and cell death is lower. Using metformin to shut down the mitochondrial respiratory chain and drive faster glucose consumption ramps up the rate of death of activated macrophages by *C. albicans* with the knock-on effect of increasing mortality. In contrast, boosting local glucose levels by continuous administration of glucose improved these outcomes [123].

Glucose depletion not only leads to the rapid death of activated macrophages but also causes inflammasome activation by activating NLRP3, due to increased fungal burden [124]. NLRP3 has a protective role during infection, being a PRR that triggers processing and secretion of IL-1α. Therefore, the regulation of NLRP3 is crucial during *C. albicans* infection [125]. In a recent study, the mechanism behind NLRP3 activation during infection of macrophages was investigated, showing that inflammasome activation was broadly uniform among multiple clinical isolates of *C. albicans*, and rather than being dependent on hyphal formation, was purely down to glucose competition. Notably, reducing fungal ability to consume glucose (by using the *tye7Δ/Δgal4Δ/Δ* mutant strains) or increasing the glucose levels both reduce NLRP3 activation and IL-1β production [124].

It was believed for a long time that hyphae are essential for the pathogenicity of *C. albicans* during infections. This hypothesis, however, was challenged with the discovery that metabolic adaptation during systemic infections can be as important as morphological plasticity [126]. In a murine model of systemic candidiasis using the yeast-locked *eed1Δ/Δ* mutant, virulence was retained, leading to rapid yeast proliferation, and higher fungal loads in organs such as the kidneys or liver. Phenotypic analyses of the mutant strain showed enhanced growth rates in physiologically relevant carbon sources, including lactate, acetate, and citrate. A few genes involved in carboxylic acid and citrate metabolism were upregulated, alongside with *GAT1* that promotes proliferation in casamino acid rich environments. Therefore, the metabolic flexibility of *C. albicans* yeast-locked *eed1Δ/Δ* mutant in using alternative carbon sources (such as fatty acids, carboxylic and amino acids) at lower concentrations or the absence of glucose enhances its colonization ability and pathogenicity. Hence, metabolic adaptation and fitness of *C. albicans* during infection not only supress the activity and recognition of immune cells, but also enhance the pathogenicity and mortality in systemic infection independently of hyphal formation.

### 4.2. Role of Metabolism during C. albicans Interactions with Epithelial Cells

While metabolic changes in immune cells have been the subject of recent studies, our current knowledge of these changes in epithelial cells (ECs) following microbial infection is limited. ECs are not merely passive barriers to prevent the invasion of microbes at the body’s exterior surfaces but are also important in maintaining the balance with resident microbial communities. There are few studies showing metabolic reprogramming in ECs during microbial infection, namely increased glycolytic activity during *Staphylococcus aureus* infection of skin keratinocytes [35].

Concerning ECs interactions with fungi, oral epithelial cells (OECs) have been found to upregulate metabolic reprogramming-related genes in response to fungal infections, including HIF1-α pathway during *C. albicans* oropharyngeal candidiasis in mice [127] or *C. parapsilosis* infection of human OECs [128]. Similar to what is observed in phagocytosed cells, *C. albicans* upregulates gluconeogenesis, the glyoxylate pathway and FAO in the late phase of interaction with OECs, which might be related to the invasion process [129]. However, further analyses should be performed to unravel the mechanisms underlying these metabolic shifts.

Additionally, vaginal EC responses to varied species of *Candida* (*C. albicans*; *C. glabrata*; *C. parapsilosis*; and *C. tropicalis*) have also been studied using dual RNA sequencing in a time course infection model for vaginal ECs, analysing both fungal and host transcriptomic profiles [130]. In this study, Pekmezovic and co-workers showed a biphasic response to *Candida* spp. in vaginal ECs. The initial response is highly uniform among *Candida* species and characterised by mitochondrial-associated type 1 interferon (IFN) signalling. Of note, most mitochondrial genes were upregulated in the early phase of *Candida* infection, and the morphology of mitochondria changed in response to the infection. Moreover, mitochondrial DNA (mtDNA) and ROS are released into the vaginal ECs cytoplasm in all *Candida* species, both acting as damage-associated molecular patterns (DAMPs). In terms of fungal transcriptome, at 3 h post-infection *C. albicans* and *C. glabrata* upregulated carbohydrate catabolic processes and stress response pathways, whilst *C. parapsilosis* upregulated, among others, genes related to amino acid metabolism, iron transport, ribosome assembly and translation. In contrast, *C. tropicalis* differentially expressed genes were mainly related to RNA processing, ribosome biogenesis and ergosterol biosynthetic processes. Unlike the early responses, the late damage-associated epithelial transcriptional response is morphology-dependent, with the hyphal-associated toxin candidalysin enhancing the host responses [130].

## 5. Conclusions and Future Perspectives

Nutritional environment and metabolic adaptations in both host and fungal cells are key during their interactions. Further characterising and understanding host immunometabolic responses to *Candida* infections will potentially help developing novel therapeutic strategies to modulate these responses. In addition, identifying which metabolic enzymes are essential during the activation of anti-*Candida* immunity will lead to the detection of genetic variants associated with higher susceptibility in individuals suffering from recurrent or chronic fungal infections. Likewise, modulating nutrients in the infection environment could help enhance host responses and/or hamper fungal growth. Therefore, further research on these promising fields must be carried out to expand our knowledge and design new strategies to tackle fungal infections.

## Data Availability

Not applicable.
